# Combined analysis of miR-200 family and its significance for breast cancer

**DOI:** 10.1038/s41598-021-82286-1

**Published:** 2021-02-03

**Authors:** Andrea Fontana, Raffaela Barbano, Elisa Dama, Barbara Pasculli, Michelina Rendina, Maria Grazia Morritti, Valentina Melocchi, Marina Castelvetere, Vanna Maria Valori, Sara Ravaioli, Sara Bravaccini, Luigi Ciuffreda, Paolo Graziano, Evaristo Maiello, Massimiliano Copetti, Vito Michele Fazio, Manel Esteller, Fabrizio Bianchi, Paola Parrella

**Affiliations:** 1 Fondazione IRCCS Casa Sollievo della Sofferenza, UO di Biostatistica, San Giovanni Rotondo, FG Italy; 2 Laboratorio Di Oncologia, Fondazione IRCCS Casa Sollievo della Sofferenza, Viale Padre Pio, 71013 San Giovanni Rotondo, FG Italy; 3 Cancer Biomarkers Lab, ISBREMIT, Institute for Stem-Cell Biology, Regenerative Medicine and Innovative Therapies, Fondazione IRCCS Casa Sollievo della Sofferenza, San Giovanni Rotondo, FG Italy; 4 Fondazione IRCCS Casa Sollievo della Sofferenza, UO di Oncologia, San Giovanni Rotondo, FG Italy; 5 Fondazione IRCCS Casa Sollievo della Sofferenza, UO di Anatomia Patologica, San Giovanni Rotondo, FG Italy; 6 Istituto Scientifico Romagnolo per lo Studio e la Cura dei Tumori (IRST) IRCCS, Meldola, Italy; 7 Fondazione IRCCS Casa Sollievo della Sofferenza, UO di Chirurgia Senologica, San Giovanni Rotondo, FG Italy; 8 Josep Carreras Leukaemia Research Institute (IJC), Badalona, Barcelona, Catalonia Spain; 9 Centro de Investigación Biomédica en Red Cáncer (CIBERONC), Madrid, Spain; 10 Physiological Sciences Department, School of Medicine and Health Sciences, University of Barcelona, Barcelona, Catalonia Spain; 11 Institució Catalana de Recerca I Estudis Avançats (ICREA), Barcelona, Catalonia Spain

**Keywords:** Cancer, Molecular biology

## Abstract

While the molecular functions of miR-200 family have been deeply investigated, a role for these miRNAs as breast cancer biomarkers remains largely unexplored. In the attempt to clarify this, we profiled the miR-200 family members expression in a large cohort of breast cancer cases with a long follow-up (H-CSS cohort) and in TCGA-BRCA cohort. Overall, miR-200 family was found upregulated in breast tumors with respect to normal breast tissues while downregulated in more aggressive breast cancer molecular subtypes (i.e. Luminal B, HER2 and triple negative), consistently with their function as repressors of the epithelial-to-mesenchymal transition (EMT). In particular miR-141-3p was found differentially expressed in breast cancer molecular subtypes in both H-CSS and TCGA-BRCA cohorts, and the combined analysis of all miR-200 family members demonstrated a slight predictive accuracy on H-CSS cancer specific survival at 12 years (survival c-statistic: 0.646; 95%CI 0.538–0.754).

## Introduction

With five highly conserved miRNAs (i.e. miR-141, miR-200a, miR-200b, miR-200c and miR-429), the miR-200 family is one of the most frequent groups of miRNAs whose expression is altered in cancer. Two gene clusters located at different chromosomes code for miR-200a/miR-200b/miR-429 (at chr1p36) and for miR-200c/miR-141 (at chr12p13). miR-200b, miR-200c and miR-429 share an almost identical seed sequence “AAUACUG”, while the seed of miR-200a and miR-141 differentiates from the other members for only one nucleotide “AACACUG”^1^. The expression regulation of the miR-200 family was associated with the *i)* suppression of EMT and tumor metastases through the miR-200/ZEB1-2 axis^2^, *ii)* inhibition of cancer stem cell self-renewal and differentiation^3^, and *iii)* reversal of chemoresistance^4^. Despite comprehensive literature that largely described the molecular function of miR-200 family, the precise role of these miRNAs in cancer has not yet completely understood, with some reports suggesting more prevalent oncosuppressive roles while other studies some possible oncogenic functions. Similar inconsistencies are also evident in few studies evaluating miR-200 family expression in tissues and their potential role as prognostic biomarkers^5^. For example, low miR-200b/c expression in breast cancer was correlated with death^3–5^, whereas high miR-200a expression was correlated with the development of distant metastases^6^. By comparing miRNA expression in normal breast tissue, in situ carcinoma, non-metastatic and metastatic breast cancers, Sánchez-Cid et al. also found that miR-200a/b were increased in metastatic tumors as compared to non-metastatic cancer^7^.

These contrasting results led us to perform an extensive expression analysis of the entire miR-200 family in two large cohorts of breast cancer patients, the first collected in our hospital (H-CSS cohort, N = 283) and the second from The Cancer Genome Atlas Breast Invasive Carcinoma (TCGA-BRCA cohort, N = 451), in order to clarify the extent of miR-200 family deregulation in breast cancer, and its specific association with clinicopathological parameters.

## Results

### Patients and treatment

Table 1 summarizes the clinicopathological information obtained from the review of medical records and descriptive statistics for the 287 enrolled cases (H-CSS cohort). Metastases at diagnosis were present in 16 cases; among non-metastatic patients (N = 271), 55 experienced disease progression (Incidence Rate, IR of 3.5 events per 100 person-years), and 30 of them died for the disease (IR of 1.7 events per 100 person-years). The median time to disease progression was 69.8 months (IQR: 33.8–107.5), whereas the overall follow-up time was 75.1 months (IQR: 40.6–109.7). Hormone receptor positive breast tumors were defined as cases expressing estrogen (ER) or progesterone (PgR) receptors in ≥ 1% of neoplastic cells as indicated by international guidelines^8^, and *HER2* status assessment was carried out according to standard recommendations^9^. Cases were staged according to the World Health Organization staging system version 7th^10^. The surrogate molecular classification was performed as described by Pasculli et al.^11^. Overall, 104 cases (38%) were classified as Luminal A, 96 cases (35%) as Luminal B; 34 cases (12%) were *HER2*-amplified, and 38 cases (14%) were Triple Negative (Table 1). Fifteen cases were not classified because the *HER2* and or ki67 status was not reported in the medical records. All patients received breast-conserving surgery or total mastectomy, plus sentinel node biopsy or complete axillary dissection. Post-surgery treatments were performed according to the following guidelines: San Gallen, NCCN and ASCO. Recurrence was defined as evidence of loco-regional and/or distant disease over 4 months from diagnosis and after curative-intent surgical treatment.

**Table 1:** Clinicopathological characteristics of the H-CSS patient cohort (N = 287).

Variable	Category	
Age (years)	Mean ± SD	59.02 ± 13.60
	Median (IQR)	59.68 (47.39–70.54)
	Range	29.7–89.3
Tumor dimension (cm)	Mean ± SD	2.97 ± 1.65
	Median (IQR)	2.5 (2.0–3.5)
	Range	0.5–11
Ki67	Mean ± SD	35.69 ± 23.16
	Median (IQR)	30 (18–50)
	Range	1–95
Tumor history—N(%)	Primitive	282 (98.26)
	Recurrence	5 (1.74)
Menopause—N(%)	No	86 (29.97)
	Yes	201 (70.03)
Histotype—N(%)	NST	261 (90.94)
	NST + ILC	5 (1.74)
	ILC	21 (7.32)
Site—N(%)	Bilateral	2 (0.7)
	Right	136 (47.39)
	Left	149 (51.92)
Stage (WHO 7)—N(%)	Stage I	43 (14.98)
	Stage IIa	99 (34.49)
	Stage IIb	45 (15.68)
	Stage IIIa	22 (7.67)
	Stage IIIb	30 (10.45)
	Stage IIIc	32 (11.15)
	Stage IV	16 (5.57)
Histological grade—N(%)	Missing values	30
	1	27 (10.51)
	2	125 (48.64)
	3	105 (40.86)
Estrogen receptor—N(%)	Negative	70 (24.39)
	Positive	217 (75.61)
Progesterone receptor—N(%)	Negative	87 (30.31)
	Positive	200 (69.69)
HER2neu—N(%)	Missing values	7
	AMP	65 (23.21)
	NEG	215 (76.79)
Surrogate molecular classification—N(%)	Missing values	15
	HER2-amplified	34 (12.5)
	Luminal A-like	104 (38.24)
	Luminal B-like	96 (35.29)
	Triple Negative	38 (13.97)

### Selection of TCGA-BReast invasive CAncer (TCGA-BRCA) cohorts

We selected a cohort of 1053 women with breast cancer not treated with neoadjuvant therapy from the TCGA data portal (https://portal.gdc.cancer.gov/). All tumors had available expression profile for all the five miRNAs of the miR-200 family. The log2 read counts were used for miRNA expression analysis. The TCGA-BRCA cohort was harmonized with the H-CSS cohort by using a two-step approach (Supplemental Fig. 2 and Supplemental Table 1): (i) the TCGA-BRCA cohort was limited to those histotypes (NST and ILC) and stages (I-IIa/b, IIIa/c and IV) represented in H-CSS (N = 822); (ii) we performed a random disproportionate sampling to align the distribution of histotypes and stages between the two cohorts; weights were overall based on H-CSS distribution, with the exception that we reduced the weights for late stages not to deplete the final cohort’s size extensively. We ultimately selected 451 patients for all subsequent analyses. Characteristics of these cohorts are reported in Supplemental Table 1.

Analysis of variance, run through a general linear model, was performed to evaluate the association of miRNAs expression and clinicopathological features, considering both main effects and interaction terms. Cox regression model was implemented to estimate hazard ratios for overall survival, defined as the time from the date of tumor resection until death from any cause. We also downloaded miRNA expression data for 104 available normal breast tissues from TCGA-BRCA data portal. Forty-eight of these normal samples were matched to 48 tumor counterparts among the cohort of 451 women considered.

### miR-200 family expression in tumor samples as compared with normal tissues

**Figure 1:**
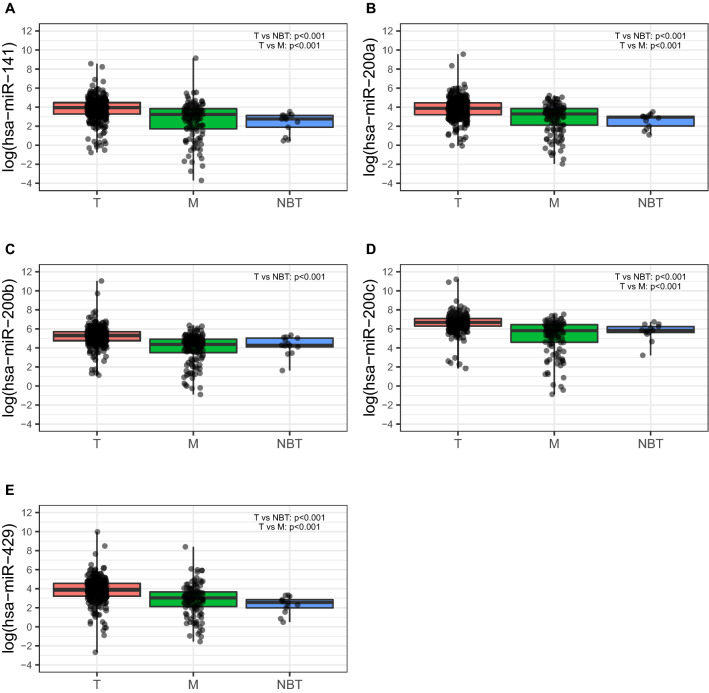
miR-200 family members expression in H-CSS cohort in tumor tissues (T), normal breast tissues distant from tumor (M) and normal breast tissue from reductive mammoplasty (NBT). Plots were performed using the R Foundation for Statistical Computing (version 3.6, packages: ggplot2, gridExtra).

**Figure 2:**
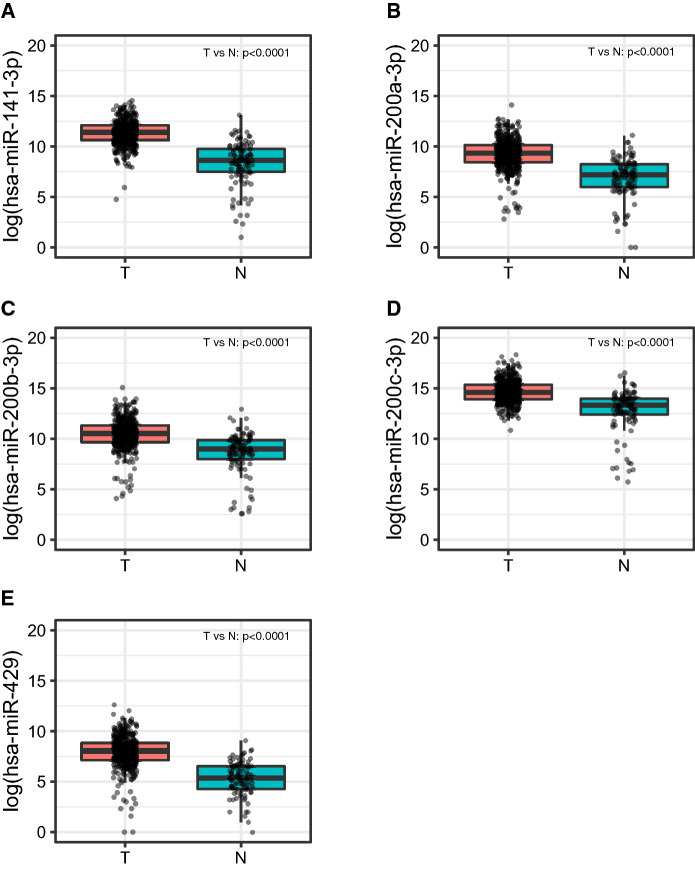
miR-200 family members expression in TCGA-BRCA cohort in tumor tissues (T), and normal breast tissues distant from tumor (N). Plots were performed using the R Foundation for Statistical Computing (version 3.6, packages: ggplot2, gridExtra).

Following evaluation of RNA quality, 283 out of the 287 samples from the H-CSS cohort showing an RNA Integrity Number (RIN) > 7.0 were suitable for the analysis (Supplemental Fig. 1). Thus, the expression profile of the entire miR-200 family could be performed in 283 breast cancers, and in 13 normal breast tissues (NBTs) obtained from reductive mammoplasty. As shown in Supplemental Table 2A and Fig. 1, all miRNAs were significantly overexpressed in tumors (*p* < 0.001) when compared to NBTs. Furthermore, almost all miR-200 family members (except for miR-200b-3p) were overexpressed in tumors as compared to normal tissue adjacent to tumor (Margin) (Supplemental Table 2B). Accordingly, in the TCGA-BRCA cohort, we confirmed the overexpression of miR-200 family in breast tumors vs. normal breast tissues (*p* < 0.0001) (Supplemental Table 3A and Fig. 2) and in matched tumor-normal pairs (N = 48; Supplemental Table 3B).

### Association among miR-200 family expression and tumor clinicopathological features

**Figure 3:**
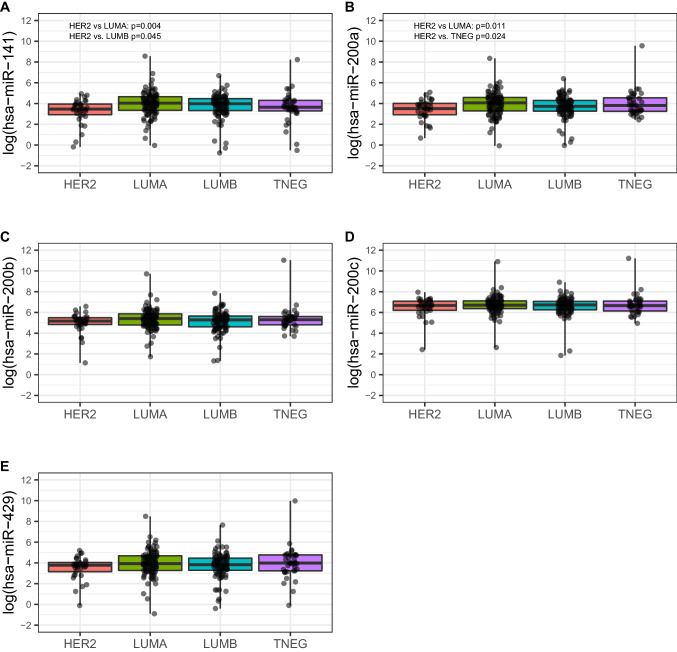
miR-200 family members expression within the surrogate molecular classification subgroups identified in the H-CSS cohort: HER2 amplified tumors (HER2), Luminal A (LUMA), Luminal B (LUMB), Triple Negative (TNEG). Plots were performed using the R Foundation for Statistical Computing (version 3.6, packages: ggplot2, gridExtra).

**Figure 4:**
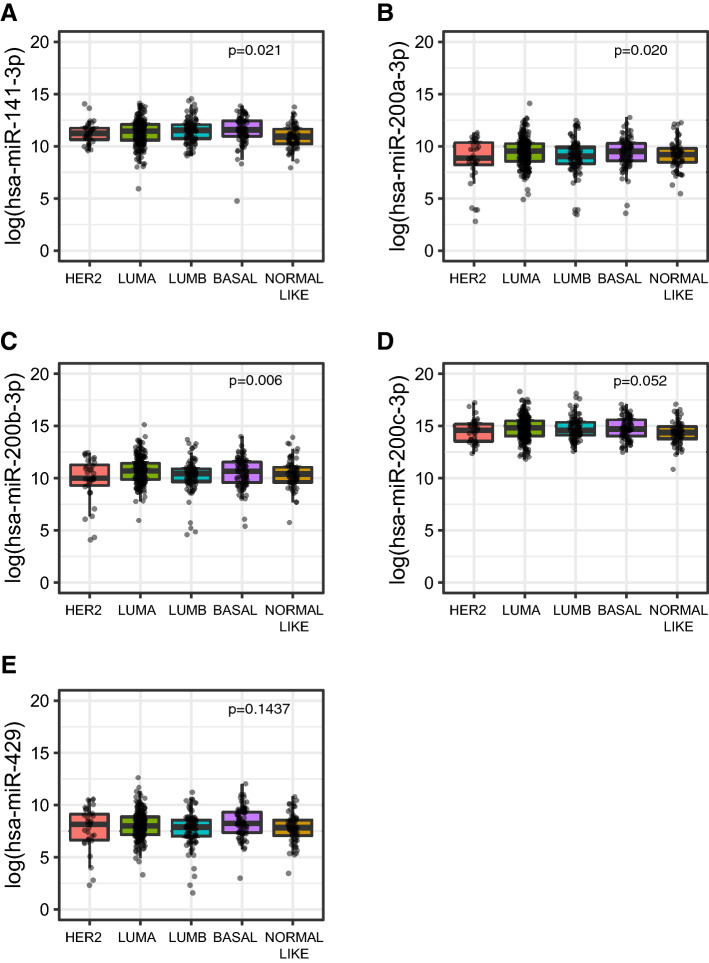
miR-200 family members differential expression within the intrinsic molecular classification subgroups in the TCGA-BRCA cohort: HER2 enriched tumors (HER2), Luminal A (LUMA), Luminal B (LUMB), Basal like, and NORMAL LIKE tumors. Plots were performed using the R Foundation for Statistical Computing (version 3.6, packages: ggplot2, gridExtra).

Next, we analyzed the correlation of miR-200 family expression with the clinicopathological characteristics in both the H-CSS (Table 2) and TCGA-BRCA cohorts (Supplemental Table 4A). In the H-CSS cohort (Table 2), the analysis across breast cancer (BC) molecular subtypes (Luminal A, Luminal B, *HER2*-amplified, and basal/Triple-negative (TNEG); see “Methods”) showed a lower expression of miR-141-3p (*p* = 0.0306) and miR-200a-3p (*p* = 0.0381) in those tumors associated to more aggressive subtypes (e.g. LUMB, HER2-amplified and TNEG) Table 2). In particular, miR-141-3p was less expressed in *HER2*-amplified and TNEG tumors, while miR-200a-3p was less expressed in *HER2*-amplified, Luminal B and TNEG subtypes (Fig. 3). Consistently, in the TCGA-BRCA cohort, we found that miR-141-3p was downregulated in the Normal-like subgroup (*p* = 0.0210) while miR-200a-3p was downregulated in *HER2-*amplified and Luminal B tumors (*p* = 0.0200). In addition, miR-200b-3p was found down regulated in the *HER2*-amplified subgroup (*p* = 0.0060) (Supplemental Table 4A; Fig. 4).

**Table 2:** Association between miRNAs and clinicopathological variables.

miRNA	Clinical variable	Category	N	Statistic#	*p* value*
miR-141-3p	Age	–-	283	r = 0.016	0.7948
	Tumor dimension	–-	283	r = 0.035	0.5616
	Ki67	–-	263	r = − 0.128	**0.0379**
	Menopause	No	85	38.61 (26.49–76.48)	0.0961
		Yes	198	53.51 (25.9–93.03)	
	Tumor Histotype	NST	257	50.63 (27.35–81.07)	0.7499
		NST + ILC	5	34.39 (28.98–48.48)	
		ILC	21	56.83 (20.8–109.97)	
	Site	Bilateral	2	98.04 (56.14–139.94)	0.4226
		Right	134	49.71 (26.49–89.27)	
		Left	147	52.02 (25.9–78.94)	
	Estrogen receptor	Negative	69	36.69 (20.98–65.17 )	**0.0146**
		Positive	214	53.84 (28.01–93.37 )	
	Progesterone reeceptor	Negative	85	37.95 (24.14–65.88 )	**0.0352**
		Positive	198	54.05 (28.05–94.51 )	
	Her2neu	Amplified	64	44.64 (26.2–72.35 )	0.1379
		Negative	212	52.98 (27.05–91.71 )	
	Stage (WHO 7)	Stage I	41	57.35 (39.33–88.36)	**0.0370**
		Stage IIa	98	42.55 (23.1–78.4)	
		Stage IIb	45	50.77 (28.05–65.08)	
		Stage IIIa	22	43.66 (17.62–54.23)	
		Stage IIIb	30	65.2 (28.74–128.88)	
		Stage IIIc	31	45.74 (24.65–80.52)	
		Stage IV	16	80.58 (29.82–189.07)	
	Histological grade	1	26	47.56 (28.74–103.33)	0.1401
		2	124	53.66 (28.52–97.22)	
		3	103	38.61 (23.1–72.73)	
	Surrogate molecular classification	HER2 amplified	33	31.94 (18.63–51.91)	**0.0306**
		Luminal A	104	56.99 (28.4–105.55)	
		Luminal B	93	53.51 (27.72–87.22)	
		Triple Negative	38	38.32 (26.74–73.94)	
hsa-miR-200a	Age	–-	283	0.028	0.6335
	Tumor dimension	–-	283	0.005	0.9334
	Ki67	–-	263	− 0.105	0.0893
	Menopause	No	85	43.13 (24.73–75.42)	0.2043
		Yes	198	50.8 (24.68–90.39)	
	Tumor Histotype	NST	257	48.76 (25.46–84.75)	0.5089
		NST + ILC	5	35.79 (25.72–36.42)	
		ILC	21	44.65 (19.03–166.08)	
	Site	Bilateral	2	147.61 (94.3–200.92)	0.3743
		Right	134	42.36 (25.72–76.84)	
		Left	147	53.33 (23.69–86.71)	
	Estrogen receptor	Negative	69	33.37 (21.48–78.68 )	0.2981
		Positive	214	51.77 (26.26–86.71 )	
	Progesterone receptor	Negative	85	44.17 (23.02–76.59 )	0.4315
		Positive	198	51.02 (26.36–90.39 )	
	Her2neu	Amplified	64	40.18 (23.21–69.3 )	0.1009
		Negative	212	52.97 (24.7–95.68 )	
	Stage (WHO 7)	Stage I	41	44.93 (23.02–85.32)	**0.0011**
		Stage IIa	98	36.49 (20.96–67.7)	
		Stage IIb	45	55.65 (28.99–85.68)	
		Stage IIIa	22	38.81 (21.48–78.68)	
		Stage IIIb	30	56.9 (33.36–161)	
		Stage IIIc	31	44.17 (27.28–80.1)	
		Stage IV	16	105.65 (36.9–204.02)	
	Histological grade	1	26	58.14 (25.72–96.78)	0.2894
		2	124	52.57 (26.27–87.14)	
		3	103	43.13 (19.23–67.7)	
	Surrogate molecular classification	HER2 amplified	33	33.37 (18.44–55.43)	**0.0381**
		Luminal A	104	58.18 (26.86–97.92)	
		Luminal B	93	41.71 (26.19–73.5)	
		Triple Negative	38	45.24 (25.72–94.59)	
hsa-miR-200b	Age	–-	283	− 0.031	0.6072
	Tumor dimension	–-	283	0.039	0.5088
	Ki67	–-	263	− 0.095	0.1226
	Menopause	No	85	196.75 (116.38–279.00)	0.7207
		Yes	198	200.15 (112.31–301.69)	
	Tumor histotype	NST	257	197.42 (111.71–289.32)	0.4229
		NST + ILC	5	222.86 (175.98–233.38)	
		ILC	21	219.44 (119.66–569.52)	
	Site	Bilateral	2	242.05 (204.54–279.55)	0.7886
		Right	134	190.94 (106.23–273.31)	
		Left	147	201.83 (126.28–330.57)	
	Estrogen receptor	Negative	69	178.88 (119.12–266 )	0.5246
		Positive	214	203.34 (111.71–319.75 )	
	Progesterone receptor	Negative	85	178.88 (113.62–264.23 )	0.5849
		Positive	198	204.09 (112.31–323.37 )	
	Her2neu	Amplified	64	199.09 (115.02–273.7 )	0.3643
		Negative	212	200.37 (113.41–307.4 )	
	Stage (WHO 7)	Stage I	41	182.02 (119.66–279)	0.0684
		Stage IIa	98	188.17 (108.48–268.28)	
		Stage IIb	45	248.55 (135.31–330.57)	
		Stage IIIa	22	177.43 (75.74–365.43)	
		Stage IIIb	30	224.37 (142.77–476.98)	
		Stage IIIc	31	203.04 (113.62–267.86)	
		Stage IV	16	235.35 (134.93–511.01)	
	Histological grade	1	26	180.52 (91.35–301.69)	0.5903
		2	124	202.44 (110.15–338.3)	
		3	103	194.95 (113.2–264.23)	
	Surrogate molecular classification	HER2 amplified	33	176.36 (126.28–242.73)	0.1588
		Luminal A	104	224.15 (120.3–352.84)	
		Luminal B	93	197.15 (101.09–285.24)	
		Triple Negative	38	199.11 (123.11–272.04)	
hsa-miR-200c	Age	–-	283	− 0.033	0.5780
	Tumor dimension	–-	283	0.044	0.4606
	Ki67	–-	263	− 0.068	0.2738
	Menopause	No	85	781.21 (572.53–1125)	0.5383
		Yes	198	814.66 (534.54–1237.5)	
	Tumor histotype	NST	257	793.15 (556.83–1191.88)	0.6936
		NST + ILC	5	786.62 (768.82–1068.29)	
		ILC	21	992.52 (542.84–1486.69)	
	Site	Bilateral	2	1831.45 (1475.34–2187.57)	0.3406
		Right	134	790.84 (522.03–1216.31)	
		Left	147	794.84 (562.58–1191.88)	
	Estrogen receptor	Negative	69	781.21 (452.92–1194.55 )	0.6537
		Positive	214	804.54 (561.25–1216.31 )	
	Progesterone receptor	Negative	85	768.82 (452.92–1194.55 )	0.6473
		Positive	198	808.92 (572.53–1210.46 )	
	Her2neu	Amplified	64	881.74 (604.47–1286.37 )	0.9829
		Negative	212	787.79 (550.85–1187.66 )	
	Stage (WHO 7)	Stage I	41	744.36 (576.41–1119.53)	0.2718
		Stage IIa	98	791.57 (528.96–1232.86)	
		Stage IIb	45	867.48 (626.76–1227)	
		Stage IIIa	22	796.29 (420.18–1128.67)	
		Stage IIIb	30	878.03 (595.54–1411.15)	
		Stage IIIc	31	773.83 (562.58–1092.21)	
		Stage IV	16	787.49 (527.48–1355.04)	
	Histological grade	1	26	667.19 (488.12–1097.21)	0.4365
		2	124	805.55 (572.36–1167.01)	
		3	103	812.15 (574.36–1229.08)	
	Surrogate molecular classification	HER2 amplified	33	791.69 (497.88–1194.55)	0.5229
		Luminal A	104	811.76 (582.91–1229.93)	
		Luminal B	93	836.18 (595.54–1175.58)	
		Triple Negative	38	780.99 (465.26–1205.38)	
hsa-miR-429	Age	–-	282	− 0.05	0.4004
	Tumor dimension	–-	282	0.046	0.4451
	Ki67	–-	262	− 0.049	0.4313
	Menopause	No	85	47.03 (28.57–75.29)	0.6537
		Yes	198	49.1 (24.94–99.57)	
	Tumor histotype	NST	257	47.03 (25.41–91.93)	0.4162
		NST + ILC	5	48.48 (45.43–87.35)	
		ILC	21	55.37 (19.03–108.22)	
	Site	Bilateral	2	89.15 (15.14–163.17)	0.9737
		Right	134	44.61 (24.94–87.71)	
		Left	147	50.75 (25.44–99.57)	
	Estrogen receptor	Negative	69	46.69 (22.94–87.71 )	0.4817
		Positive	214	49.6 (26.91–93.37 )	
	Progesterone receptor	Negative	85	46.26 (23.28–87.71 )	0.4184
		Positive	198	49.97 (27.2–99.57 )	
	Her2neu	Amplified	64	44.54 (26.16–84.92 )	0.2805
		Negative	212	50.42 (25.42–102.58 )	
	Stage (WHO 7)	Stage I	41	48.48 (22.94–83.93)	**0.0078**
		Stage IIa	98	41.94 (22.92–66.79)	
		Stage IIb	45	58.16 (32.02–90.82)	
		Stage IIIa	22	46.48 (28.81–136.24)	
		Stage IIIb	30	77.09 (37.76–152.36)	
		Stage IIIc	31	42.25 (28.57–76.99)	
		Stage IV	16	97.96 (37.67–169.11)	
	Histological grade	1	26	48.99 (23.28–104.25)	0.2133
		2	124	49.97 (26.34–101.45)	
		3	103	44.39 (25.41–74.27)	
	Surrogate molecular classification	HER2 amplified	33	43.79 (23.52–56.63)	0.4289
		Luminal A	104	50.68 (26.34–106.23)	
		Luminal B	93	45.73 (24.94–85.39)	
		Triple negative	38	53.89 (25.41–117.92)	

^#^ In case of continuous clinical variables (i.e. age, tumour dimension and Ki67), r denotes Pearson correlation coefficient with log-transformed miRNA expression whereas in presence of categorical clinical variables, median along with interquartile range (IQR, i.e. first-third quartiles) of the miRNA expression was reported.

*p values from Pearson correlation or two-sample *t* test (or ANOVA model as appropriate) using log-transformed miRNA expressions was reported for continuous and categorical variables, respectively. *p*-value <0.05 are reported in bold.

In the H-CSS cohort, miR-141-3p, miR-200a-3p and miR-429 expression was increased in advanced stage disease (stage IV) (*p* = 0.037, *p* = 0.0011 and *p* = 0.0078 respectively) (Table 2). In the TCGA cohort, miR-200a-3p (*p* = 0.0128), miR-200b-3p (*p* = 0.0009) and miR-200c-3p (*p* = 0.0013) were increased in invasive lobular carcinoma (Supplemental Table 4A). To evaluate whether these differences may affect the association with molecular subtypes, we performed a multivariable analysis adjusting for stage, histotype and molecular subtype. Overall, our results indicate that miR-200a-3p and miR-141-3p remain significantly associated with the molecular subtypes in breast cancer after the adjustments in the H-CSS cohort (Table 3), whereas an association with miR-141-3p and miR-200c-3p was found in the TCGA cohort (Supplemental Table 4B).

**Table 3:** Univariate and multivariable analysis of the association between miRNAs and clinicopathological characteristics in the TCGA cohort.

H-CSS COHORT		Univariate analysis—*p* value*	Multivariable analysis—*p* value*
miR-141-3p	Histotype	0.7499	0.8906
	Stage	**0.0370**	0.2186
	Surrogate Molecular Classification	**0.0306**	**0.0233**
miR-200a-3p	Histotype	0.5089	0.6958
	Stage	**0.0011**	**0.0318**
	Surrogate molecular classification	**0.0381**	**0.0235**
miR-429	Histotype	0.4162	0.6375
	Stage	**0.0078**	0.0864
	Surrogate molecular classification	0.4289	0.1134

*p-values <0.05 are reported in bold.

**Table 4:** Prognostic accuracy of each outcome-specific weighted miRNA score at median and maximum time horizons.

Outcome	Time horizon (years)	N.events/total	Survival c-statistic (95%CI*)
Cancer specific survival (CSS)	7 (median)	27/263	0.650 (0.535–0.766)
	12 (max)	29/263	0.646 (0.538–0.754)
Progression free survival (PFS)	7 (median)	45/263	0.590 (0.497–0.682)
	12 (max)	53/263	0.528 (0.402–0.654)
Distant metastases free survival (MFS)	7 (median)	43/259	0.613 (0.527–0.699)
	12 (max)	52/258	0.572 (0.479–0.664)

*95% confidence interval after 1000 perturbation-resamplings of the data.

### Evaluation of miR-200 family prognostic value in breast cancer cases

The association with time-to-event outcomes (i.e. CSS, DFS, and MFS) was evaluated in the H-CSS cohort without metastases at diagnosis and with complete information about survival outcomes (Supplemental Fig. 1). As shown in Supplemental Tables 5 and 6, tumor dimension, stage, hormone receptor status, *HER2-*amplification, and surrogate molecular classification were associated with DFS, MFS, and CSS, while high Ki67 was associated with DFS and MFS only. No statistically significant associations were found with any miRNA of miR-200 family in the overall population. Next, we investigated the prognostic role of the miR-200 family in the TCGA-BRCA cohort by considering the subgroup of patients without metastases at diagnosis (cohort C1, N = 435; Supplemental Fig. 2). We scored 56 deaths and a median follow-up for surviving women of 2.4 years (Q1 = 1.2; Q3 = 4.6 years). Although this cohort showed a shorter follow-up and limited number of events, we were able to confirm the prognostic role of stage, estrogen and progesterone receptor status, *HER2* status, and molecular subtypes (Supplemental Table 7A). In line with the results obtained in the H-CSS, we did not observe a statistically significant association of any of the miR-200 family members with overall survival (OS) in multivariate analysis (Supplemental Table 7B). These figures were confirmed in the larger cohort of N = 806 subjects without metastases (cohort B1, Supplemental Fig. 2), scoring 101 events, and with a follow-up length (median = 2.4 years; Q1 = 1.2; Q3 = 4.7) comparable to the smaller C1 cohort (Supplemental Table 7A, B).

**Table 5:** Estimated regression coefficients used to compute multiple weighted miRNA scores.

Outcome	miRNA (log expressions)	coefficients (weights)	*p* value
Cancer specific survival (csS)	hsa-miR-141	− 0.77834	0.0073
	hsa-miR-200a	0.77455	0.0495
	hsa-miR-200b	− 1.22087	0.0433
	hsa-miR-200c	0.71390	0.1173
	hsa-miR-429	0.23035	0.4661
Progression free survival (pfs)	hsa-miR-141	− 0.50129	0.0170
	hsa-miR-200a	0.57881	0.0540
	hsa-miR-200b	− 0.78914	0.0730
	hsa-miR-200c	0.48904	0.1450
	hsa-miR-429	0.09213	0.6720
Distant metastases free survival (mfs)	hsa-miR-141	− 0.52081	0.0110
	hsa-miR-200a	0.44872	0.1270
	hsa-miR-200b	− 0.90106	0.0410
	hsa-miR-200c	0.66320	0.0470
	hsa-miR-429	0.12025	0.5670

Last, we evaluated whether the combined expression of miR-200 family members was able to predict survival outcomes. As shown in Table 4, when all miRNAs were jointly considered for the building of the weighted scores, only a slight predictive accuracy on H-CSS outcome at 12 years was found (survival c-statistic: 0.646; 95%CI 0.538–0.754). Regression coefficients (weights) used to calculate the scores were reported in Table 5.

## Discussion

In the attempt to elucidate the extent of miR-200 family deregulation in breast cancer and, hence, its potentiality as clinically significant biomarker, we profiled a large series of breast cancer cases with a long follow-up (H-CSS cohort) and the TCGA-BRCA cohorts. First, we found in both H-CSS and TCGA-BRCA cohorts that the global miR-200 family expression is increased in tumors as compared with normal breast tissue or margin. Since miR-200 family is mainly expressed in epithelial cells, these results are most likely due to the enrichment in fibrous connective adipose tissue typical of the normal breast. The enrichment in normal breast epithelial component might also explain some inconsistencies among literature data. Consistently with our data, Amorim et al.^12^ found an increased expression of miR-200b-3p and miR-141-3p in tumors as compared with margin, whereas other studies reported reduced expression of miR-200b-3p^3^ and miR-200c-3p^5^ in tumors as compared with margins.

In both H-CSS and TCGA-BRCA cohorts, we found a differential expression of miR-200 family members within the molecular subgroups identified by the surrogate molecular classification (H-CSS cohort) and intrinsic molecular subtypes (TCGA-BRCA cohort). In particular, lower expression of miR-141-3p/miR-200a-3p was associated with *HER2*-amplified, Luminal B, and Triple Negative (H-CSS cohort) or Normal Like (TCGA-BRCA cohort) breast cancer subtypes. This is consistent with reports describing that miR-200 family loss of expression unleashes *ZEB1* expression^13^, which in turn induces epithelial-to-mesenchymal transition (EMT), which is an important step forward in the initial phase of the metastatic spreading from the primary tumor.

Functionally speaking, the association between miR-200 family and metastatic processes have been widely investigated in different tumor types, including breast cancer and, once again, conflicting results have been reported. Indeed, the ectopic expression of miR-200a and miR-200b was shown to inhibit EMT features in undifferentiated, non-tumorigenic breast cancer cells, and impair proliferation, migration, and invasion in triple negative breast cancer^14^. Accordingly, miR-200c/141 cluster deletion affects breast cancer stem cell heterogeneity by promoting the generation of EMT-like stem cells, which resulted in increased tumor metastasis^3^. miR-200 family members were also found to support Epidermal Growth Factor (EGF)-driven invasion, with the miR-200bc/429 cluster showing stronger effects than the miR-200a/141 cluster^1,15^. Moreover, miR-200a suppressed cell proliferation in breast cancer by targeting mitochondrial transcription factor A^16^, and impaired EMT-like transformation, thus migration, by regulating SIRT1 in breast epithelial cells^17^. Nevertheless, while these studies likely suggest a tumor suppressor role for miR-200 family members, others indicate that higher expression of miR-200 family members might induce rather than prevent metastases formation. For instance, the forced expression of miR-200a/miR-200b in MCF10 mammary cells induced an enhanced epithelial program, aldehyde dehydrogenase (ALDH) activity, mammosphere growth and ability to form branched tubuloalveolar structures while promoting orthotopic tumor growth and lung colonization in vivo, suggesting that miR-200 family members may promote traits of highly proliferative breast luminal progenitor cells^7^. Likewise, miR-200c/141 cluster overexpression induced by SerpinB2 was shown to foster breast cancer cell metastasis^18^. Furthermore, miR-200a overexpression was found to enhance malignant transformation of immortalized human mammary epithelial cells^19^, to protect tumor cells from apoptosis, and promote metastases^4^ and chemoresistance^20^. Altogether, these discrepancies lead to hypothesize that the biological functions of miR-200 family members may depend on the cellular context, tumor molecular subtype, and stage of tumor progression^21^.

In our study, the association between miR-200 family expression and patients’ outcome was evaluated in terms of DFS, MFS, and CSS in the H-CSS cohort including 283 non-metastatic breast cancer cases with a median follow-up of 75 months. In the TCGA-BRCA cohort, only overall survival data were available instead. Our analyses did confirm the prognostic role of lymph node status, estrogen and progesterone receptors status, *HER2* status, and molecular subtypes in both H-CSS and TCGA-BRCA cohorts. However, we did not observe any statistically significant association of the miR-200 family members with patients’ outcome in multivariable analyses. Indeed, in the H-CSS cohort, the combined expression of miR-200 family members only showed a slight predictive accuracy on CSS outcome at 12 years (Table 4).

**Figure 5:**
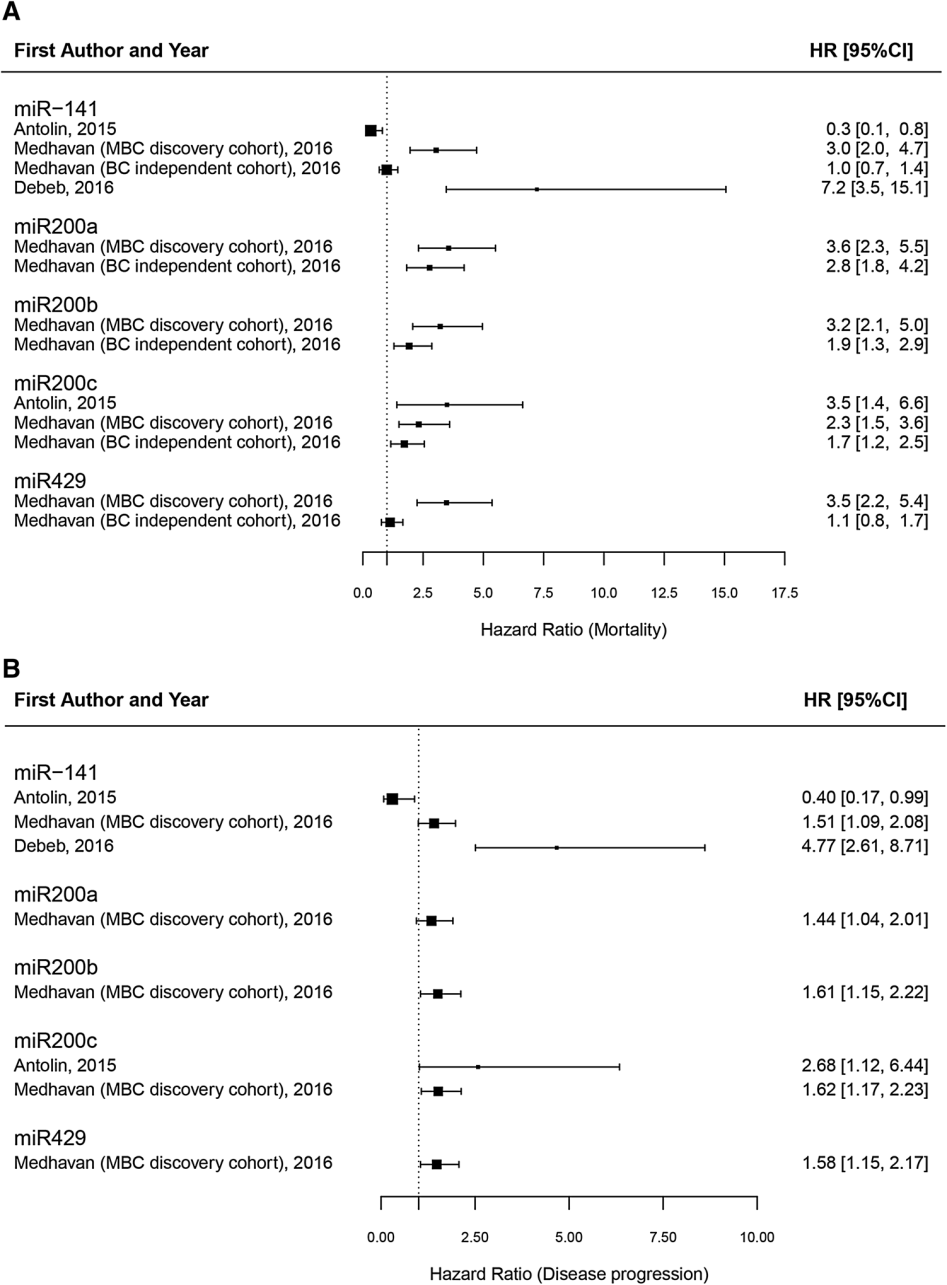
Forest plot of hazard ratios (HR) for studies on plasma samples from breast cancer patients. Plots were performed using the R Foundation for Statistical Computing (version 3.6, packages: ggplot2, gridExtra).

To date, only a minority of studies^22^ have performed the expression analysis of miR-200 family members in breast cancer tissues, and evaluated its association with patients’ outcomes (Fig. 5, and Supplemental Table S8). Among those, only one study reported an hazard ratio of 0.231 (95%CI 0.094–0.564) in univariable analysis for miR-200c in a patient cohort including only luminal tumors^12^. Other three studies^23–25^ evaluated the association between miR-200 family members expression in plasma samples and patient’s outcome (Supplemental Table S8). In particular, Medhavan et al.^24^ found an association between increased expression of miR-200a, miR-200b and miR-200c and higher risk of overall mortality in univariable analyses (Fig. 5).

## Conclusion

To the best of our knowledge, this is the first study evaluating the expression of all miR-200 family members in breast cancer tissues in order to identify potential combination biomarkers of clinical relevance. Our results suggest a differential expression of miR-200 family in breast cancer as compared to normal breast, and within the breast cancer molecular subgroups identified by either surrogate classification (H-CSS cohort) or intrinsic molecular classification (TCGA-BRCA cohort). Nevertheless, the correlation analyses with breast cancer patients’ prognosis exclusively found a weak predictive accuracy of the combined expression of miR-200 family on CSS outcome at 12 years in the H-CSS cohort. Although these results seem not to encourage the use of miR-200 family members as combination biomarkers in breast cancer, we cannot rule out that such a role might be held within a single breast cancer subgroup. Indeed, in the H-CSS cohort the number of cases and event outcome is not sufficient for subgroup analyses, whereas only partial information about overall survival and no data on progression are available within the TCGA-BRCA cohort. Thus, this possibility needs to be further investigated in studies specifically designed to evaluate miR-200 family expression in each of the breast cancer subtypes.

## Materials and methods

### Study design, setting and eligibility criteria

This study is part of the project TRANSCAN Joint Transnational Call (JTC) 2013-BREMIR initiated in 2015 at the Fondazione IRCCS Casa Sollievo della Sofferenza (H-CSS), aimed to identify novel biomarkers predicting disease progression and metastases development in breast cancer patients. In this study, we evaluated the miR-200 family expression in a retrospective consecutively collected cohort of 287 breast cancer cases (H-CSS cohort) with a median age of 60 years (Supplemental Table 1).

We conducted the study according to the REporting of tumor MARKer Studies (REMARK) guidelines^8,26^, and a prospectively written research (TRANSCAN-BREMIR) plan. Breast cancer tissues were collected between January 2006 and December 2014 at the Breast-Unit, Fondazione IRCCS Casa Sollievo della Sofferenza.

Following pathological evaluation, tissue samples were snap-frozen in liquid nitrogen and stored at − 80 °C. For legal reasons, only women older than 18 years of age with tumors greater than 1.0 cm in diameter were included in the study. For each sample, a 5 μm hematoxylin/eosin stained section was visually inspected by light microscopy to select tumor areas with at least 70% viable cancer cells rather than normal specimens, obtained from reductive mammoplasty, to check for the absence of tumor cells among normal epithelial. The study methodologies using these samples were carried out following the international of Helsinki Declaration 7th revision (2013, EU Directive 2004/23/EC) and the Italian (D. Lgs. 30/06/2003, n. 196) regulations for research on human subjects. All experimental procedures of this study were approved by the Ethical Committee of the Fondazione IRCCS Casa Sollievo della Sofferenza (Prot N 140/CE). A written informed consent was obtained from all patients following the experimental protocol approved by the Ethical Committee.

### RNA isolation and RT-qPCR analysis

RNA was isolated from H-CSS tissue samples by Trizol reagent (Invitrogen) according to the manufacturer’s instructions. Total RNA concentration was determined by the absorbance measurement at 260 nm and 280 nm using the NanoDropTM 1000 spectrophotometer (Thermo Fisher Scientific). The RNA quality and integrity were analyzed through 2100 Expert Analyzer (Agilent Technology), and only RNAs with RIN (RNA Integrity Number) ≥ 7.0 were considered acceptable. Then, 10 ng of total RNA was reverse transcribed to single stranded cDNA by using TaqMan MicroRNA Reverse Transcription Kit (Thermo Fisher Scientific) and 5 × specific stem-loop RT primers for both individual miR-200 family members and the endogenous control, according to the manufacturer’s instructions. RT positive and negative controls were included in each batch of reactions. To assess miR-200 family expression levels in the H-CSS cohort, we applied a relative quantification method with a standard curve^27^. The expression levels of each miR-200 family member were assessed by using TaqMan MicroRNA Assays that were as follows: hsa-miR-200a-3p, assay ID: 000502; hsa-miR-200b-3p, assay ID: 002251; hsa-miR-200c-3p, assay ID: 002300; hsa-miR-141-3p, assay ID: 000463; hsa-miR-429, assay ID: 001024, and normalized to RNU48 endogenous control, assay ID: 001006 (Thermo Fisher Scientific).

Each qPCR run was performed by using 0.5 μl of TaqMan microRNA (20X), 5 μl of TaqMan Universal PCR Master Mix II, No AmpErase UNG, and 1 μl of cDNA. The PCR conditions were as follows: at 95 °C for 10 min, followed by 40 cycles (95 °C for 15 s, 60 °C for 1 min). All samples were run in triplicates. Each plate included positive and negative controls of reverse transcription and multiple water blanks. qPCR reactions were performed on ABI PRISM 7900HT Sequence Detection System and the SDS 2.4 software (Thermo Fisher Scientific) was used for post-run analyses. For each miR-200 family member and RNU48 control, standard curves were constructed by plotting the threshold cycle (Ct) values against log10 of the copy number, and fitting by linear least square regression. For each sample, miR-200 family member expression was determined as the ratio of any single miR-200 family member’s copy number to the RNU48 copy number. Then, it was multiplied by 1000 for more straightforward tabulation (i.e. miRNA target/RNU48) × 1000)^27^.

### Statistical analysis

Patients’ clinicopathological characteristics were reported as median along with interquartile range (IQR, i.e. first-third quartiles) or frequencies and percentages for continuous and categorical variables, respectively. Normal distribution assumption of miRNA expression was evaluated by Q-Q plots and Shapiro-Wilks test, and a log-normal distribution for all miR-200 family members was detected. The two-sample *t* test (or ANOVA model as appropriate) was used to assess comparisons of log-transformed miRNA expression among patient groups. Pearson correlation coefficient was estimated to assess the correlation between natural log of miRNA expression and continuous variables. Time-to-event analyses were performed by univariable and multivariable proportional hazards Cox regression models and risks were reported as Hazard Ratios (HR) along with their 95% Confidence Interval (95%CI).

The individual overall follow-up time was defined as the time between the enrollment date (i.e. at the time of snap-frozen fresh tissue collection) and the occurrence of the death due to cancer (Cancer Specific Survival, CSS), whereas the individual time to tumor progression or distant metastasis was defined as the time between the enrollment date and the occurrence of the first disease progression (Disease Free Survival, DFS), or the first distant metastasis (Metastasis Free Survival, MFS). For patients who did not experience any event as above, their individual follow-up time was defined as the time between the enrollment date and the end of the observational period (i.e. last available examination).

Furthermore, annual mortality and disease progression rates were defined as the number of events divided by the number of person-years × 100. When each miRNA expression was considered as the main covariate into a univariable Cox model, HRs were reported with respect to patients groups defined by miRNA’s median value (i.e. above vs. below the median). Moreover, multivariable Cox models were also performed with the inclusion of lymph node and surrogate molecular classification as further covariates. A weighted miRNA score was computed for each survival outcome at issue by the assessment of a multivariable Cox model, which included all miRNAs (natural log of expression) of the miR-200 family as main covariates. Weighted scores were calculated as the linear combination of the regression coefficients by the value of each miRNA (natural log of expression). The prognostic accuracy of each miRNA score was assessed at 7 (i.e. the median time horizon) and at 12 years (i.e. the maximum time horizon) by survival C-statistic, along with its 95% CIs derived following 1000 perturbation-resampling^28^. A two-sided *p* value < 0.05 was considered for statistical significance. All statistical analyses were performed using SAS Release 9.4 (SAS Institute, Cary, NC, USA). Plots were performed using the R Foundation for Statistical Computing (version 3.6, packages: ggplot2, gridExtra).

### Ethics approval and consent to participate

The study methodologies using human samples were carried out following the international of Helsinki Declaration 7th revision (2013, EU Directive 2004/23/EC) and the Italian (D. Lgs. 30/06/2003, n. 196) regulations for research on human subjects. All experimental procedures of this study were approved by the Ethical Committee of the IRCCS Casa Sollievo della Sofferenza (Prot N 140/CE). The informed consent was obtained from all patients following the experimental protocol approved by the Ethical Committee.

## Supplementary Information

Supplementary Legends

Supplementary Figure 1.

Supplementary Figure 2.

Supplementary Tables.

## Data Availability

The datasets analysed during the current study are available from the corresponding author on reasonable request.
