# Supplementary material for: Combined analysis of miR-200 family and its significance for breast cancer
Source: Sci Rep. 2021 Feb 3;11:2980. doi: 10.1038/s41598-021-82286-1 (PMC7859396; doi:10.1038/s41598-021-82286-1)
Supplement: Supplementary file 1 — Supplementary Legends [file 41598_2021_82286_MOESM1_ESM.docx]

**Supplemental Figure 1.** Diagram showing inclusion criteria for survival analyses in the H-CSS cohort. Plots were performed using the R Foundation for Statistical Computing (version 3.6, packages: ggplot2, gridExtra).

**Supplemental Figure 2.** Diagram showing the selection of TCGA-BRCA cohorts. Plots were performed using the R Foundation for Statistical Computing (version 3.6, packages: ggplot2, gridExtra).
