# Supplementary figures and images for: Combined analysis of miR-200 family and its significance for breast cancer
Source: Sci Rep. 2021 Feb 3;11:2980. doi: 10.1038/s41598-021-82286-1 (PMC7859396; doi:10.1038/s41598-021-82286-1)

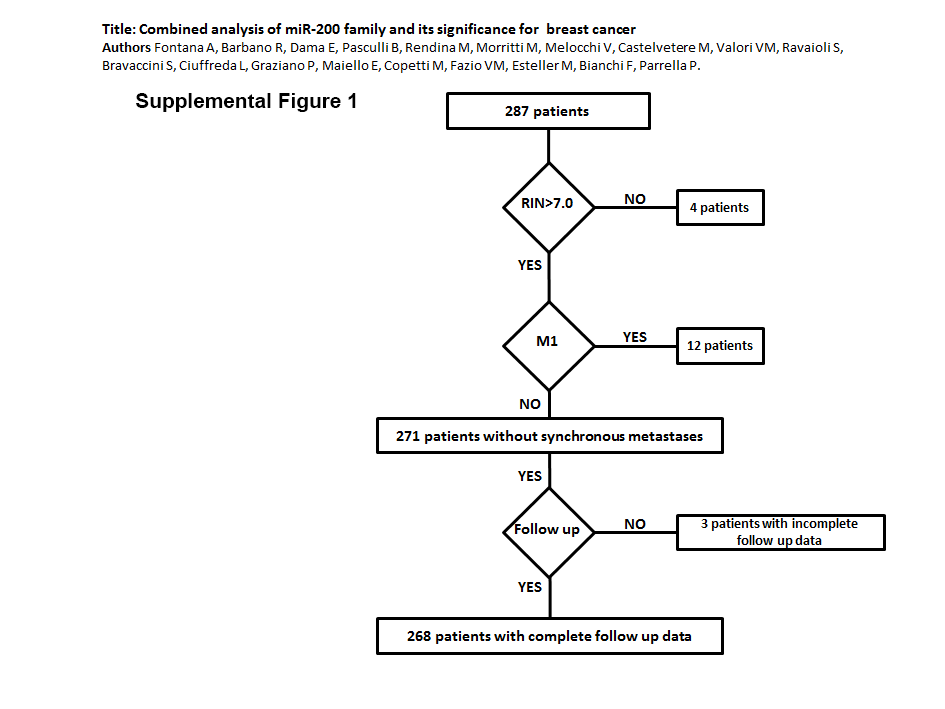

Supplement: Supplementary file 2 — Supplementary Figure 1. [file 41598_2021_82286_MOESM2_ESM.tif]

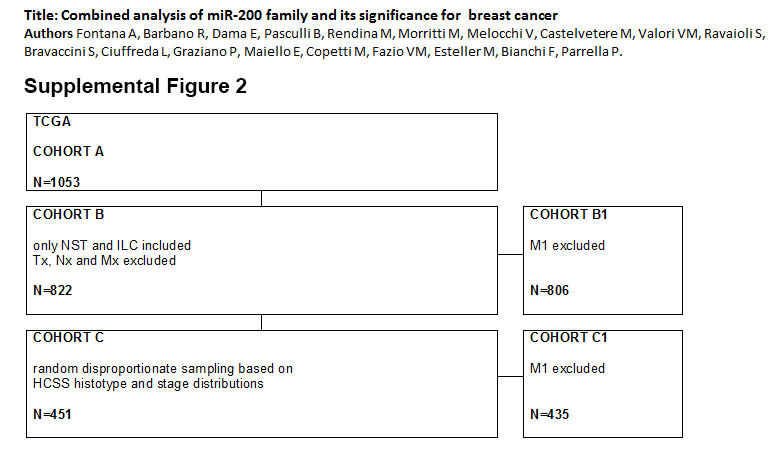

Supplement: Supplementary file 3 — Supplementary Figure 2. [file 41598_2021_82286_MOESM3_ESM.tif]
